# Toxic Metals and Non-Communicable Diseases in HIV Population: A Systematic Review

**DOI:** 10.3390/medicina57050492

**Published:** 2021-05-13

**Authors:** Opeyemi M. Folorunso, Chiara Frazzoli, Ifeyinwa Chijioke-Nwauche, Beatrice Bocca, Orish E. Orisakwe

**Affiliations:** 1 African Centre of Excellence for Public Health and Toxicological Research (ACE-PUTOR), University of Port Harcourt, PMB, Port Harcourt 5323, Rivers State, Nigeria; folorunso.opeyemi@uniport.edu.ng; 2 Department for Cardiovascular, Endocrine-Metabolic Diseases, and Aging, Istituto Superiore di Sanità, 00162 Rome, Italy; chiara.frazzoli@iss.it; 3 Department of Clinical Pharmacy, Faculty of Pharmacy, University of Port Harcourt, Port Harcourt 5323, Rivers State, Nigeria; ifeyinwa.chijioke-nwauche@uniport.edu.ng; 4 Department of Environment and Health, Istituto Superiore di Sanità, 00161 Rome, Italy; beatrice.bocca@iss.it; 5 Department of Experimental Pharmacology & Toxicology, Faculty of Pharmacy, University of Port Harcourt, Port Harcourt 5323, Rivers State, Nigeria

**Keywords:** heavy metals, toxicity, comorbidities, AIDS

## Abstract

*Background and Objectives:* HIV has been a serious global health concern since its discovery, with about 37.9 million people living with HIV worldwide as of 2018. Sub-Saharan Africa (SSA) accounts for 68% of the infection and contributed 74% of the 1.5 million deaths in 2013 despite having only 12% of the total world population residing in the region. This systematic review has attempted to determine the association between heavy metal toxicity and the occurrence of non-communicable diseases in the HIV/AIDS population. *Materials and Methods:* Three databases were systematically searched: PubMed, Scopus, and Google Scholar for studies written in English and published between 1 April 2000 and 12 April 2020. Studies were excluded if the main outcomes were not measured or did not meet the inclusion criteria. *Results:* All the six included studies are cross-sectional in design, and therefore were evaluated using the STROBE checklist. The data extraction was done using an extraction table; the ratio of female to male participants included in the study was 1.09:1. Qualitative analysis was used due to the heterogeneity in the heavy metal biomarkers and the outcome measured by the included studies. Two studies compared the concentration of heavy metals in HIV-positive and HIV-negative participants while one compared the levels between HAART-naïve and HAART-treated participants, and three determined the association between heavy metal toxicity and non-communicable diseases (liver fibrosis, anaemia, and reproductive parameters, respectively) in HIV-positive patients. *Conclusions:* Blood lead, cadmium, and mercury levels were higher in HIV-seropositive than -seronegative subjects, whereas serum zinc level was lower in HIV-seropositive than -seronegative subjects, but the causal association between heavy metals and non-communicable diseases in HIV subjects is largely unknown. Interdisciplinary research between nutrition, toxicology, and human health is envisaged for primary and secondary prevention and treatment.

## 1. Introduction

Human immunodeficiency virus (HIV), since its discovery, has been a serious global health concern [1]. As of the end of 2018, 37.9 million people were living with HIV worldwide. Though 12% of the total world population resides in Sub Sahara, the region accounts for 68% of the infection and contributed to 74% of the 1.5 million AIDS-related deaths in 2013 [2,3]. There has been a reduction in new HIV infections and mortality across the Sub-Saharan countries but prevalence is still high due to the emergence of widespread coverage of combined antiretroviral therapy (cART) [2,3]. This has improved the life expectancy of HIV-positive persons despite the absence of a cure, thereby transforming HIV infection into a chronic disease with the predisposition to the development of comorbidities, namely hypertension, diabetes mellitus, chronic renal disease, chronic pulmonary diseases, liver disease, and cancer [4].

The pathophysiology underlying the development of these non-communicable diseases in the HIV population include immunosuppression, antiretroviral drug toxicity, HIV-related inflammation, and hyper-coagulation [4,5,6]. Comorbidities are discovered from studies to be more common and manifesting at an earlier age in persons living with HIV than in HIV-uninfected matched controls [7,8]. Since the introduction of ART, chronic kidney diseases have surpassed opportunistic infections as the major cause of chronic morbidity in HIV-positive patients [9]. This has necessitated the recommendation of a specific protocol for the management of chronic renal diseases in HIV-infected patients [9]. In the past years, many inflammatory markers have been studied in association with clinically evident cardiovascular disease (CVD) and carotid intima-media thickness (CIMT) in HIV-infected patients to ascertain increased cardiovascular risk observed in HIV infection [10]. Ongoing studies have established the presence of comorbidities associated with chronic HIV infection: HIV-associated CVD [11], HIV-associated renal and genitourinary diseases [5], HIV-associated malignancies [12], HIV-associated non-Hodgkin lymphoma (NHL) [13], HIV-associated endocrine dysfunction [14], and HIV-associated pulmonary diseases [4]. These comorbidities lead to avoidable, untimely death that blunts the health gains attained in HIV-infected populations [6].

HIV infects the central nervous system (CNS), causing neuronal damage and loss prior to the development of neurologic symptoms [15]_._ The infected cells within the CNS are thought to cause the subsequent release of chemokines and cytokines that further disrupt the blood-brain barrier. This encourages invasion by increasing the number of inflammatory cells that may be responsible for the neurotoxicity and development of HIV-associated neurocognitive disorder (HAND) [16,17]. The notion that HIV-infected individuals age faster than the general population has also been proposed as a probable cause of the increased prevalence of HAND in that population [18]. The occurrence of HAND has also been attributed to mitochondrial toxicity of some forms of cART, especially the Non-reverse transcriptase inhibitors (NRTI) [19]. HIV infection and its treatment have also been found to increase the risk of endocrine dysfunction, resulting in increased morbidity and mortality from DM, CVD, and thyroid dysfunction [14]. Highly active antiretroviral therapy (HAART) (protease inhibitors and non-nucleoside reverse transcriptase inhibitors) was discovered to increase levels of testosterone and 17beta-estradiol [20].

Despite studies (e.g., [21,22,23,24,25]) confirming the contribution of heavy metal toxicity on the development of NCDs in the general population [26,27,28], there remains a paucity of information on the impact of environmental pollution on the development of NCDs in the HIV-positive population.

The impact of the early exposure to heavy metals on the immune system of children is manifested as susceptibility to immune-related diseases [21]. Fe is an important essential element involved in various physiological activities, but its ability to form free radicals when in the free state through the Fenton reaction leads to the production of reactive oxygen species (ROS). The ROS further attack proteins, lipids, and enzymes, resulting in eventual oxidative stress and damage to the cells [22]. An increase in Fe accumulation in tissues and elevated ferritin levels was discovered as HIV infection progresses [23]. Pb and Hg also inhibit glutathione peroxidase, which gives rise to oxidative stress that elicits inflammatory responses and can also dysregulate Ca^2+^ homeostasis, causing protein and cell damage [24]. Toxicity starts from the production of free radicals, leading to oxidative stress, which in turn damages enzymes, proteins, lipids, and DNA, resulting in damage to various organs, neurotoxicity, and carcinogenesis [25].

This paper seeks to express how much has been done with respect to the impact and contribution of heavy metals on the development of NCDs in the HIV-positive population.

## 2. Materials and Methods

### 2.1. Search Strategy and Information Sources

Following the Preferred Reporting Items for Systematic Reviews and Meta-Analyses (PRISMA) guidelines, a thorough search was performed using three databases: PubMed, Scopus, and Google Scholar. Search terms used included the following: (HIV positive individual) OR (Human Immunodeficiency Virus positive individuals) OR (AIDS patients) OR (Acquired Immune Deficiency Syndrome patients) OR (HIV positive patients) OR (PLWHIV/AIDS) OR (people living with HIV/AIDS) AND (cadmium toxicity) OR (cadmium poisoning) OR (chromium toxicity OR (chromium poisoning) OR (lead toxicity) OR (lead poisoning) OR (plumbism) OR (saturnism) OR (iron poisoning) OR (iron toxicity) OR (iron overload) OR (acquired haematochromatosis) AND (elevated biomarkers) OR (elevated TNF-alpha) OR (elevated IL-6) OR (elevated IFN-gamma) OR (elevated 8-OHdG) OR (higher level of TNF-alpha) OR (higher level of IL-6) OR (higher level of IFN-gamma) OR (higher level of 8-OHdG) AND (carcinogenesis) OR (cancer) OR (malignancy) AND (cell damage) OR (DNA damage) OR (cell death) OR (apoptosis) AND (neurotoxicity) OR (neuro toxic effects) OR (elevated serum Pb) OR (elevated serum Lead) OR (elevated serum cadmium) OR (elevated serum Cd) OR (elevated serum iron) OR (elevated serum Fe) OR (elevated serum chromium) OR (elevated serum Cr).

### 2.2. Eligibility Criteria

**Figure 1:**
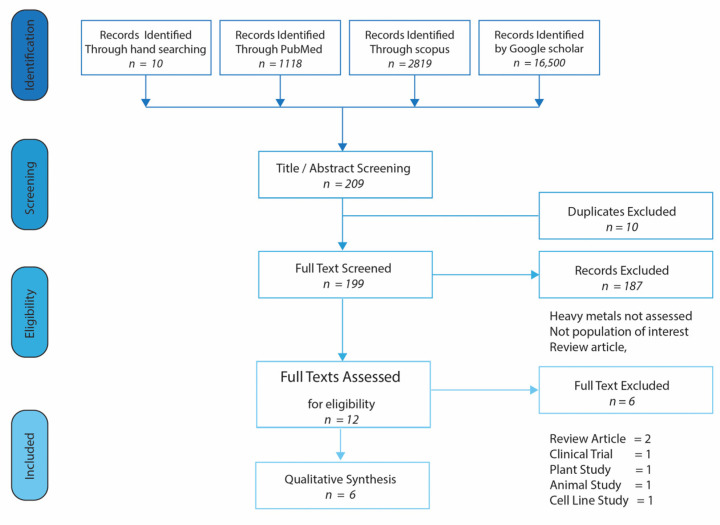
Flow Chart of Included Studies for Systematic Review.

We included observational, comparative clinical trials and case-control studies written in English and published between 1 January 1990 and 13 April 2020. Papers were included if they reported on: (1) adult population >18 years, (2) human studies, (3) HIV/AIDS subjects, (4) heavy metal toxicity, and (5) biomarkers of heavy metal toxicity and non-communicable diseases. The exposure of interest is heavy metal toxicity (Cadmium, or Cd; Lead, or Pb; Iron, or Fe; Mercury, or Hg; Chromium, or Cr; and Nickel, or Ni), while the outcome is HIV-infected individuals showing elevated biomarkers (TNF-alpha, IL-6, CRP, IFN-gamma, 8-OHdG, serum Pb, serum Hg, serum Cr, serum Fe) and co-morbidities (cardiovascular disease, type 2 DM, cancer, chronic kidney disease). Flow diagram of the literature search and study selection is described in Figure 1.

### 2.3. Study Quality Assessment

Two independent reviewers (OMF and OEO) conducted a full-text review for eligibility. Mendeley library version 1803 was used to store the identified studies from which 10 duplicates were discovered and removed.

### 2.4. Data Extraction and Synthesis

Data extraction was conducted using an extraction table. The table includes the following characteristics of the included studies: the name of the first author, the year of publication, the country in which the research took place, study design, sample type, markers assessed, method of the assessment, statistical analysis used, and number, sex, age, ethnicity, marital status, level of education, and employment status of participants. All the six included studies are cross-sectional studies, so they were evaluated using the STROBE checklist. Qualitative synthesis was conducted based on the heterogeneity in the heavy metal biomarkers and outcomes measured by the included studies. Two-tailed tests were done and *p* < 0.05 was considered significant for all statistical comparisons.

### 2.5. Critical Appraisal

**Figure 2:**
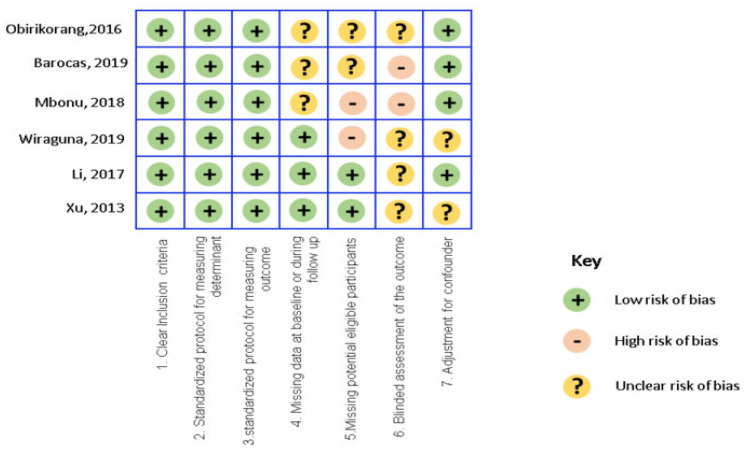
Risk of Bias Assessment.

The selected studies were critically appraised on seven items: clarity of inclusion criteria, standardization of measurement of determinant, standardization of measurement of outcome, missing data at baseline or follow-up; missing data on potential eligible participants, and blinding and adjustment for confounders. Bias risk was assigned as low risk, high risk, or unclear using an adapted Cochrane collaboration tool. A risk-of-bias summary is presented in Figure 2.

## 3. Results

The studies independently measured the concentration of heavy metal biomarkers in HIV patients. Two of the articles made a comparison of the concentration of heavy metals in HIV-positive with HIV-negative participants, while two articles compared heavy metal levels between HAART-naïve and HAART-treated participants, and two studies were done in the HIV population without controls.

Efforts were made to determine the association between heavy metal toxicity and NCDs (liver fibrosis, anaemia, and reproductive parameters) in HIV-positive patients. None of the studies measured the major biomarkers of interest, TNF-alpha, IL-6, and IFN-gamma 8-OHdG, but heavy metals (Pb; Cd; Hg; Ni; Zinc, or Zn; Iron, or Fe) were measured.

Most of the studies were at risk of sampling bias as none of the studies calculated the sample size, while only two reported the type of sampling technique used, i.e., consecutive [29] and multistage [30] sampling. This limits the external validity of their findings and the generalizability of their results. Two of the articles engaged further analysis: exploratory [31] and sensitivity [32].

### 3.1. Characteristics of Included Studies

Six studies involving 12,679 participants (Table 1) examined heavy metal concentration in association with HIV status, HAART-treated, HAART-naive status, the occurrence of NCDs (liver fibrosis, anaemia, deranged male reproduction hormone parameters), and severity of another communicable disease (condyloma acuminata) in the HIV population. There were altogether 851 HIV-infected and 11,828 HIV-uninfected participants. Female participants were more than males: 6630 (52.2%) and 6049 (47.7%), respectively. The female to male ratio of participants included in the study was 1.09:1. The ages of the participants were between 18 and 70 years.

The studies were conducted between 2013 and 2019 and cut across the United States of America, Europe, Asia, and Africa. Only one of the studies reported on ethnicity [30], with 42.4% whites, 21.8% blacks, and 35.8% from other ethnicity groups. The level of education was reported by two studies: a greater percentage of participants in Xu et al. (2013) [30] had more than high school education (47.8%), while 27.1% and 24.9% of them had less than high school education and a high school education, respectively. The contrast is the case in the study by Obirikorang et al. (2016) [32] where most of the participants had less than high school education (28.8%), while 20.4% and 17.6% had a high school and more than high school education, respectively. This is the only study that reported the marital status of the participants, with 62.1% married and 37.9% single, and also the only study that mentioned the employment status of the participants, with the greater percentage (54.9%) engaged in informal employment while other participants were almost equally distributed between formal and unemployed (22.9% and 22.3%, respectively). Xu et al., [30] is the only study that captured the PIR of its participants. All the studies reported on the HIV status of their participants; three of the studies included only HIV positive participants [31,32,33]. Only 0.51% of participants of Xu et al. (2013) [30] were HIV-positive, while 66.7% and 40% were HIV-positive in Ma et al. (2018) [34] and Wiraguna et al. (2019) [29], respectively.

Xu et al. (2013) [30] assessed the prevalence of elevated concentrations of heavy metals in HIV-positive individuals and compared the levels with HIV-negative participants. The blood of participants were sampled for Cd, Pb, Hg, HIV antibody, and serum cotinine. Pb levels were significantly higher in HIV-infected subjects aged 18–34 (1.41, 95%CI: 0.99–2.01); those who reported being neither non-Hispanic, white, or black (1.95, CI: 1.57–2.41); and those who had only graduated from high school (1.87, CI: 1.32–2.65) compared to their HIV-negative counterparts (0.97 (0.95–1.00), 1.26 (1.21–1.31) and 1.18 (1.14–1.23), respectively); all *p* < 0.05. HIV-infected participants with PIR <1.00 had significantly higher values of blood Cd, Pb, and Hg than HIV-uninfected subjects: this was explained as the possibility of increased exposure to environmental pollutants due to the residential location of the low-income class in the community.

Similarly, Ma et al. (2018) [34] compared the heavy metal concentration in HIV-positive HAART-treated, HIV-positive HAART-naïve, and HIV-negative controls. The blood level of Pb was found to significantly decrease with increasing CD4 count. On the other hand, Li et al. (2017) [33] sampled serum plasma, urine, and semen of 50 HIV-positive men and assessed the effects of heavy metals (Pb, Cd, and Zn) on the reproductive hormones (LH, FSH, and Testosterone) of HIV-positive men. The results were analyzed using ANOVA spearman’s rank correlation: HIV-1 viral loads were significantly associated with increased seminal Pb which was also positively correlated with LH, while serum Pb was negatively correlated with FSH.

Furthermore, Obirikorang et al. (2016) [32] measured the markers of Fe homeostasis to determine the association between anaemia, disease progression, and HAART in a cohort of HIV HAART-naïve and HIV HAART-treated subjects. The results were analyzed using an unpaired *t*-test, Fisher’s exact test, and one-way ANOVA. Participants with anaemia had a significantly lower CD4/CD3 lymphocyte count. The remaining two studies measured the concentration of Zn in HIV-positive participants and made efforts to associate Zn level to occurrence and severity of other diseases (condylomata acuminata and liver fibrosis) in the HIV population. Wiraguna et al. (2019) [29] sampled the plasma of participants for HIV, Zn, and CD4 count and made a comparison between the mean Zn levels in condyloma acuminata patients with HIV and those without HIV infection. The mean Zn levels in condyloma acuminata patients with HIV were significantly lower than those without HIV infection, while Barocas et al. (2019) [31] measured the impact of Zn on the occurrence of liver fibrosis among ART-naïve young HIV/HCV co-infected persons. No significant association was found between continuous Zn level and FIB-4 score.

### 3.2. Study Findings

Six heavy metals (Pb, Cd, Zn, Hg, Fe, Ni) were analyzed in the six included studies (Table 2), with Pb and Cd assessed most frequently, both by three of the six included studies. All the studies were cross-sectional assessments that involved measurements of heavy metals in the blood (plasma or serum), semen, and urine using ICP-MS, Graphite furnace atomic absorption spectrophotometer, and Flexor XL analyzer (Fe). Four heavy metals (Pb, Cd, Hg, Ni) had significant positive associations while two (Zn and Fe) had negative associations with participants’ HIV and treatment status (Table 3).

Such heavy metals can be categorized into biological essential (Ni, Fe, and Zn) and non-biological essential (Pb, Hg, and Cd) heavy metals [35].

#### 3.2.1. Biological Essential Heavy Metals

**Zinc (Zn):** Two of the studies [29,31] investigated the relationship between Zn and the development of condyloma acuminata and advanced liver cirrhosis in HIV-positive patients, respectively, while Li et al. [33] assessed the association between semen quality of HIV-positive men and the concentration of seminal Zn. One of the studies [29] revealed that the mean plasma Zn levels were significantly lower in condyloma acuminata patients with HIV infection than those without HIV infection, with the difference in mean plasma Zn levels of 7.31 μg/dL (95% CI 2.25–12.37, *p* < 0.05). Barocas et al. [31] reported that the prevalence of advanced liver fibrosis was similar for those with Zn deficiency compared to those with normal Zn levels (27.7% vs. 23.0%, respectively). No association between Zn deficiency and advanced liver fibrosis was detected in both the adjusted regression model (aOR: 1.28, 95% CI: 0.62–2.61, *p* = 0.51) and exploratory analyses. Li et al. [33], on the other hand, discovered that the level of Zn was positively associated with semen quality as evidenced by sperm count of 216.4 + −84.6 in HIV-positive men with seminal Zn <30.7 mg/L and 308.8 + −56.8 in those with seminal Zn >30.7 mg/L.

**Iron (Fe):** One of the included studies [32] evaluated the biomarkers of Fe homeostasis in relation to anaemia, disease progression, and HAART in a cohort of HIV HAART-naïve and HAART-treated subjects. HAART-treated participants had significantly higher CD4/CD3 lymphocyte counts, Hb, heamatocrit, MCV, MCH, RDW-SD, serum Fe ferritin, and transferrin saturation (*p* < 0.05), while WBC count, serum transferrin, and TIBC were, however, higher among HIV-naïve participants (*p* < 0.05). Anaemic participants were 23.8%, who also had significantly lower CD4/CD3 lymphocyte count (*p* < 0.0001) and lower mean of red cell indices (HCT, *p* < 0.0001; MCV, *p* < 0.0001; MCH, *p* < 0.0001; MCHC, *p* = 0.0017). Moreover, serum Fe (*p* = 0.0019), ferritin (*p* =0.0021), and TSAT (*p* = 0.0002) were significantly lower in anaemic patients than in those without anaemia. Serum transferrin (*p* < 0.0001) and TIBC (*p* < 0.0001) were, however, higher in anaemic patients than in the non-anaemic. The frequency of anaemia was, however, higher among HAART-naive participants and consistent with a possible increase of disease progression. The markers of Fe stores for all participants in this study were within the normal limit. The reduced level of Fe in association with an unexplainably high level of transferrin (Fe-transporting protein) is expected in Fe deficiency [36]. The result suggested that Fe deficiency is not the cause of anaemia in HIV infection and therefore Fe supplementation is discouraged to prevent the complications of Fe overload [37].

**Nickel (Ni)**: Only one [34] of the included studies measured Ni and compared Ni concentration in HIV-positive HAART-treated and HIV-positive HAART-naïve participants. The result revealed significantly higher mean blood levels of Ni in HIV-positive patients compared to controls: (HIV-positive: 0.89 ± 1.19 μg/dL, HIV-negative: 0.11 ± 0.01 μg/dL, *p* < 0.001; HAART-naïve: 0.95 ± 1.51 μg/dL, HAART-treated: 0.84 ± 0.11 μg/dL, HIV-negative controls: 0.11 ± 01, *p* < 0.001), while blood level of Ni increased with increasing CD4 count, though not statistically significant (*p* = 0.41).

#### 3.2.2. Non-Biological Essential Heavy Metals

**Lead (Pb):** A study conducted by Xu et al. [30] in the U.S. used the National Health and Nutrition Examination Survey (NHANES) 2003–2010 to compare exposures to heavy metals, including Cd, Pb, and total Hg, in HIV-infected and non-HIV infected subjects. The study participants were 11,761 with 60 HIV-infected participants. Pb was found to be significantly higher in the blood sample of HIV-positive subjects compared to those without HIV (1.43 vs. 1.11 μg/L, *p* = 0.016). Pb was higher in HIV-infected patients aged 18–34 (neither white, black, or non-Hispanic and only graduated from high school) compared to HIV-negative patients (*p* < 0.05). Although Ma et al. [34] reported similar significantly higher levels of Pb in HIV-positive subjects compared with their negative counterparts (1.22 ± 1.00 μg/dL and 0.57 ± 0.41 μg/dL respectively, *p* < 0.001), they further discovered that the level of Pb was higher in HAART-treated than HAART-naïve or HIV-negative controls (1.07 ± 0.85, 1.38 ± 1.16 and 0.57 ± 0.41 respectively, *p* < 0.001). Li et al. [33] conducted a cross-sectional study among HIV-positive men in China. The study assessed the associations between semen quality or serum hormone and the concentration of three heavy metals (Pb, Cd, and Zn) in semen, urine, and serum. The study investigated the potential effects of heavy metals on reproductive parameters in HIV-infected men. It was reported that Pb was significantly correlated with semen quality and serum hormone in HIV-infected samples. HIV viral loads were also significantly associated with increased seminal Pb which was positively correlated with LH but negatively correlated with FSH [33]. Men with elevated seminal Pb concentrations (≥8.6 μg/L) exhibited significantly higher LH (6.8 ± 0.6 vs. 5.1 ± 0.4 mIU/mL, *p* < 0.05) and serum Zn (0.79 ± 0.08 vs. 0.56 ± 0.05 mg/L, *p* < 0.05) than those with seminal Pb < 8.6 μg/L. Men with serum Pb ≥ 6.4 μg/L exhibited significantly lower urine Zn (0.75 ± 0.08 vs. 1.14 ± 0.16 mg/L, *p* < 0.05) than those with serum Pb < 6.4 μg/L. Similarly, in-utero and postnatal exposure to Pb was associated with increased levels of immunoglobulin IgE in the cord blood and increased IgE levels among non-Hispanic white children but not in other groups of children [38,39], thus signaling the deleterious effects of early heavy metal exposure on the humoral immunity and the resultant adverse health outcomes [40].

**Cadmium (Cd):** Xu et al. [30] discovered significantly higher levels of blood Cd (0.47 vs. 0.33 μg/L, *p* = 0.005) in HIV-seropositive subjects compared to controls. A significant association was observed between HIV infection and increased blood Cd, with infected individuals having blood Cd level 1.16 times higher than HIV-uninfected individuals (CI: 1.01–1.34, *p* = 0.03). A higher blood level of Cd was found even after adjusting for pack-years of smoking. Xu et al. [30] also found that female subjects with HIV infection had higher levels of blood Cd (0.54 vs. 0.36, *p* = 0.03) and Pb (1.34 vs. 0.88, *p* = 0.03) compared to those females without HIV. The findings suggest that a higher prevalence of chronic diseases among HIV-infected patients might be a resultant effect of their significant exposure to Cd compared to non-HIV infected individuals. Additionally, impaired renal and liver functions as complications of HIV infection may lead to poor detoxification of heavy metals, resulting in their accumulation in the body [30]. Participants with elevated urine Cd (≥1.4 μg/L) exhibited significantly higher semen volume (3.1 ± 0.4 vs. 1.8 ± 0.2 mL, *p* < 0.05) and lower motile sperm count (75.6 ± 14.2 vs. 134.2 ± 20.6 ×10^6^/mL), *p* < 0.05) than those with urine Cd < 1.4 μg/L in an observational study which assessed the association between semen quality and toxic metals [33]. However, there were no significant differences in the reproductive parameters of the two groups (seminal Cd ≥ 1.7 μg/L and <1.7 μg/L, serum Cd ≥ 0.3 μg/L). Similarly, Ma et al. [34] reported results consistent with the two other studies with significantly higher levels of Cd in HIV-positive subjects when compared with their HIV-negative counterparts (0.62 ± 0.27 and 0.10 ± 0.01, respectively, *p* < 0.001). The study further established the significantly higher level of Cd in HAART-treated (0.68 ± 0.04) compared to HAART-naïve (0.55 ± 0.26) and HIV-negative control subjects (0.10 ± 0.01, *p* < 0.001). The defective aftereffect of both HIV infection and HAART treatment on the renal and liver functions of HIV HAART-treated subjects has been deduced as the cause of elevated Cd level in this population [30,34].

**Mercury (Hg):** Two studies [30,34] compared the concentration of Hg in HIV-positive and HIV-negative participants. The concentration was higher in both studies though only significantly in one of the studies [34], where the level of Hg in HIV-positive subjects doubled that of HIV-negative ones (0.08 and 0.04, respectively, *p* < 0.001). The concentration of Hg in HAART-treated subjects (0.09) was higher than HAART-naïve (0. 06) and HIV-negative controls (0.04), though not statistically significant, unlike Cd.

The study findings stratified by heavy metals are outlined in Table 3.

## 4. Discussion

Heavy metal exposure enhances the expression of factors associated with inflammation, which is the hallmark of NCDs [41]. Different biomarkers are affected by this exposure with the resultant plethora of events that lead to various diseases. Interferon-gamma (IFN-g) is an important cytokine for innate and adaptive immunity to viral infections and activator of macrophages. It also induces the expression of molecules of the major histocompatibility complex (MHC) class II, which is known to be inhibited by Pb [42,43,44]. Pb induces a shift towards Th2-like immune responses and thus lowers resistance to intracellular microbes. It has also been found to activate type 2 (Th2) responses that promote humoral immunity typified majorly by secretion of IL-4 and others, like IL-5, IL-10, and IL-13, while it inhibits Th1 responses promoting cellular cytotoxic immunity with the secretion of IL-2 and IFN-g [41,45,46,47]. Pb, by changing the ratio of Th1 and Th2 cells and stimulating B-cell activity, may have the potential to induce antibody-mediated autoimmune disorders [48]. Pb has also been found to elevate the expression of TNF-α and soluble forms of tumour necrosis factor receptors (TNF-Rs) [49]. Furthermore, the onset of atopic dermatitis in infants at six months of age was found to be associated with cord blood Cd levels. Although it is not in the focus of the present study, it is noticeable that maternal urinary arsenic (As) concentration during pregnancy is associated with the total number of infections that required a physician visit or prescription medication, as well as lower respiratory infections requiring medication in infants at four months of age [50]. Gestational and early childhood environmental exposure to As was also associated with increased death from various cancers in adults less than 50 years of age [51]. There are increased blood levels of IgA, IgG, and IgM in children less than three years of age who had postnatal Pb exposure [52]. Urinary Cd levels were also associated with dose-dependent suppression of serum IgG, but not IgM, IgA, or IgE, in children aged 5–14 years [53]. The effect of Cd exposure on cell-mediated immunity in offspring was found to be sex- and dose-dependent in a number of animal studies, while As exposure at an early stage of life also affected cell-mediated immune response in offspring in a dose-dependent manner [51].

This review assessed the association between heavy metal concentration and NCDs among HIV individuals. Six studies [29,30,31,32,33,34] were identified in our search, from which six heavy metals were checked for their contribution to the development of varied health challenges (anaemia, liver fibrosis, male reproductive hormonal imbalance) among HIV-infected individuals. A gap in knowledge was identified as most of the articles did not report on the biomarkers of interest. Six heavy metals were assessed, three of which are biologically essential (Fe, Zn, Ni), while three are non-essential (Pb, Cd, Hg) heavy metals. All the non-essential heavy metals were reported to be significantly higher in HIV-infected participants whether HAART-treated or not compared to HIV-negative controls [30,33,34]. Xu et al. [30] reported that Hg was not significantly high among HIV-positive subjects except in those with PIR < 1.00 (1.00 (0.78–1.28) and 0.70 (0.64–0.76), respectively, *p* < 0.01). Meanwhile, Ma et al. [34], the only other study that assessed Hg and who did not report on participants’ PIR, revealed not only significantly higher Hg in HIV-positive subjects but the concentration doubled that of HIV-negative subjects (0.08 and 0.04, respectively, *p* < 0.001). Although Xu et al. [30] reported high levels of all the heavy metals assessed (Pb, Hg, Cd) in HIV subjects compared to their negative counterparts, Cd was more significantly higher especially among the male gender (0.45 (0.35–0.59) and 0.32 [0.31–0.33], respectively, *p* < 0.01) and those with PIR < 1.00 (0.75 (0.49–1.13) and 0.43 (0.40–0.46), respectively, *p* < 0.01).

This result suggests more exposure of HIV-infected individuals to environmental toxicants than HIV-negative subjects, though other studies attributed this to the possible liver and renal impairment caused by HIV infection resulting in a defective detoxification process and accumulation of these toxic metals in the body [54].

Furthermore, Shen et al. [38] found that Hg and Cd chloride-induced apoptosis in heavy-metal-exposed pGL2 cells. Though different heavy metal ions selectively affect the signaling pathways, Hg chloride was found to strongly inhibit antigen-induced T-cell proliferation and directly induce T-cell death by apoptosis in resting CD4+ T lymphocytes.

The role of micronutrients like vitamin D in the absorption of both essential and toxic elements has been reported in the literature [55,56]. The absorption of biological essential metals (including Zn, Fe, and Cu) is improved at a sufficient level of vitamin D, whereas at greater concentration, it increases the absorption of toxic elements like Pb, Cd, aluminum (Al), and cobalt (Co) [55]. This has been further supported by the seasonal increase in blood Pb levels in children during summer due to the increased level of vitamin D associated with the season. Additionally, there was a disruption in the physiological functioning of vitamin D as a result of an accumulation of toxic metals in the body, as evidenced by hindering the renal synthesis of active 1,25-dihydrovitamin D [57].

Few studies have found a significant reduction in absolute number and percentage of CD3+ and CD4+ cells in individuals with Pb exposure [58], reduction in the absolute number and percentage of CD4+ cells among a group of Pb-exposed workers with a mean blood Pb level of 74.8 μg/dL compared to control [59], and decrease of CD4+ T lymphocytes in children exposed to Pb [60]. Furthermore, CD4/CD3 count was significantly lower in anaemic patients [32] while the relationship between Zn deficiency and CD4 count <200cells was established [29,61]. Ma et al. [34] reported that blood levels of Pb, Cd, and Hg were negatively correlated with CD4 count, whereas Ni was positively correlated with CD4 count, though not statistically significant. Similarly, Barocas et al. [31] found that the only variable that was significantly associated with advanced liver fibrosis was CD4 count <350 cells/μL.

Additionally, the effect of heavy metal toxicity on reproductive hormones of HIV-positive men was reported by Li et al. [33], who discovered the significant relationship between HIV viral load and increased seminal Pb. Though the effect of heavy metals on male reproduction has been established [26,33,62,63], there are a number of conflicting findings in the literature about the effect of HIV on human male reproduction. Krieger et al. discovered no abnormality in sperm count, morphology, and other seminal parameters despite HIV shedding in the semen of HIV-positive individuals on Zidovudine, as against the findings by Bujan et al. and Dulioust, who reported multiple anomaly indices of the sperms in HIV-infected patients despite adjustments for possible sources of bias [64,65,66].

Zn, though classified as heavy metal, is an important micronutrient, and its effect is rather protective than toxic [67]. The health benefits of Zn in HIV have been well documented and known to slow down the progression of the disease. The deficiency can result in declined CD4 count, poor prognosis, and increased HIV mortality [68]. Wiraguna et al. [29] reported that the concentration of serum plasma Zn was lower among HIV-positive patients with condylomata acuminata (57.27 ± 8.32 μg/dL) than in their HIV-negative counterparts (64.59 ± 8.20 μg/dL), with an average difference of 7.31 (95% CI: 2.25–12.37, *p* < 0.05). Barocas et al. [31], on the other hand, did not find any association between Zn deficiency and the occurrence of advanced liver fibrosis. This study differs from prior research [69], and the conflicting result was attributed to younger participants and many other risk factors for liver fibrosis that may limit the ability to detect a significant impact of Zn deficiency in exploratory analysis.

Obirikorang et al. [32] evaluated the prevalence of anaemia and measured markers of Fe homeostasis in relation to anaemia, disease progression, and HAART among HIV patients. Anaemia is one of the major predictors of mortality in the HIV population [70], and it is equally affected by Fe deficiency or redistribution. A total of 23.8% of the participants were anaemic, which is a rate lower than what was observed in studies done in other African countries [71,72]. This disparity was attributed to a higher proportion of HAART-treated participants in this study. The frequency of anaemia was higher among HAART-naïve patients due to an increasing viral burden, disease progression, and cytokine-mediated myelosuppression leading to anaemia. The study supported previous research as it confirmed HAART treatment as a protective tool against anaemia [70,72].

The findings from this review are pointers to the possible impact of heavy metal toxicity on immune cell count, humoral immunity, and cytokine secretion and the further decline of the health outcome in the HIV-infected population due to the underlying pathogenesis of immunosuppression. There is little literature available on the impact of heavy metals on the occurrence of NCDs in the HIV population. This made it difficult to get information about the link between heavy metals toxicity and the prevalence of NCDs in the HIV population despite what is known in the general population and the fact that NCDs occur at an early age in HIV-infected individuals. More studies are needed in this area to bridge the gap in knowledge identified.

### 4.1. Summary of Evidence and Considerations

This review revealed that measured toxic metals were higher in HIV-positive subjects whether they were on HAART or not, and of importance is the finding that toxic metals were higher in HAART-treated than HAART-naïve subjects [42]. The potential effect of heavy metal toxicity on the reproductive parameters of HIV-infected men was equally established [41], while Zn deficiency was correlated to the severity of condyloma acuminata, another communicable disease [36]. The increased prevalence of anaemia in later stages of HIV infection, especially among the HAART-naïve, was observed, which further suggests the protective effect of HAART [41].

These findings can be used to improve the diagnosis and management protocol of HIV-positive patients by ensuring regular monitoring of heavy metal levels from the point of diagnosis, which will ensure an objective correlation of the health status of the patients to their body burden of heavy metals. The need for Zn supplementation and dietary interventions should also be incorporated as a secondary prevention strategy into the treatment protocol while protecting food from pollution and contamination during handling and cooking [73]. Huge implications of the interplay between toxic exposures and nutrition should be kept in high consideration in the prevention and management of diseases [74]. Provided safety assessment, natural antidotes, or dietary probiotics could be useful against heavy metals toxicity and body burden [75,76], while the antioxidant and anti-inflammatory activity of African medicinal plants could be interesting to mitigate the risks of comorbidities [77,78]. The findings are equally an educative tool for patients on the effect of heavy metal toxicity on the prognosis of the disease state and the need to ensure compliance with their medications (HAART) and reduce heavy metal exposure. In addition, toxicovigilance systems [79] and advocacy for enforcement of government policies on environmental protection can also be achieved [80].

### 4.2. Limitations

This review identified the lack of adequate research on the impact of heavy metal toxicity on the health outcomes of people living with HIV/AIDS (PLWHIV). Most of the outcomes of interest (elevated biomarkers: TNF-alpha, IL-6, IFN-gamma, 8-OHdG) proposed in the protocol were not assessed in the included studies. Quantitative analysis was not done in this review due to the heterogeneity of the heavy metals and outcomes measured by the included studies. There are also varied statistical methods used for the analysis such as chi-square test, *t*-test, Wilcoxon, Fisher’s exact, two-tail tests, correlation coefficient, *p*-values, etc. For the same reasons, analytical procedures, e.g., from sample pre-treatment to separation, detection, and quantification (e.g., [81]), were not investigated in the present study. Additionally, few studies were found in the literature linking heavy metal toxicity to the occurrence of non-communicable diseases in PLWHIV, and all the studies included in the review are cross-sectional and lack the strength to evaluate causality. Prospective study should be the focus of future research on this subject so that the actual impact of heavy metal toxicity can be ascertained in this population.

## 5. Conclusions

The environmental impact on the HIV-associated co-morbidities remains largely unknown as the few studies included are cross-sectional and lack the strength of making causal associations. Prospective and longitudinal studies aimed at assessing the impact of exposure to heavy metals on the HIV-positive population with standard biomarker monitoring should be a major research focus because of the link between heavy metal toxicity and the development of NCDs, which may further blunt the health gains of cART in this population. The interplay between HIV, hormone-affecting antiviral therapy, and heavy metals as endocrine disruptors should also be further investigated. Interdisciplinary research between nutrition, toxicology, and human health is envisaged for the prevention and treatment of HIV and comorbidities.

## Figures and Tables

**Table 1:** Demographic Characteristics of Patients in Each Study.

Demographic Indicators	Xu et al., 2013	Obirikorang et al., 2016	Li et al., 2017	Ma et al., 2018	Wiraguna et al., 2019	Barocas et al., 2019
Number of participants	11,761	319	59	300	45	204
Country	USA	Ghana	China	Nigeria	Indonesia	Russia
Age(years)	18–49	>18	23–44	30–35	18–60	18–70
Sex(*n*%)						
Female	6184 (52.5)	217 (68)		162 (54)	17 (37.8)	50 (24.5)
Male	5577 (47.4)	102 (32)	50(100)	138 (46)	28 (62)	154 (75.5)
Ethnicity		N/A	N/A	N/A	N/A	N/A
White	4983 (42.4)			0		
Black	2567 (21.8)			300		
Other	4211 (35.8)			0		
Level of Education		N/A	N/A	N/A	N/A	N/A
<High School	3190	92 (28.8)				
High School	2939	65 (20.4)				
>High School	5624	56 (17.6)				
Abuse (Yes)	N/A	N/A	N/A	N/A	N/A	179 (88.2)
Heavy drinking						190 (93.1)
Moderate drinking						14 (6.9)
Current Cocaine Use Yes (%)						4 (2.0)
Marital Status *n* (%)	N/A		N/A	N/A	N/A	N/A
Married		198 (62.1)				
Single		121 (37.9)				
Employment Status *n* (%)	N/A		N/A	N/A	N/A	N/A
Formal		73 (22.9)				
Informal		175 (54.9)				
Unemployed		71 (22.3)				
HIV Status						
HIV	60 (0.51)	319 (100)	50 (100)	200 (66.7)	18 (40)	204 (100)
Non HIV	11,701 (99.5)	0 (0)	0 (0)	100 (33.3)	27 (60)	0

HIV, human immunodeficiency virus; USA, United States of America; N/A, not available.

**Table 2:** Characteristics of Included Studies.

Author	Study Type	Population (*n*)	Sample Type	Markers Assessed/Method	Statistical Analysis
Xu et al., 2013	Cross-sectional	HIV positive (60), HIV negative (11,701)	Blood (Serum/Plasma)	Cadmium, lead, mercury-plasma mass spectrometry, HIV antibody-enzyme immunoassay (EIA) (Bio-Rad Laboratories, Hercules, CA, USA). Western blot (Calypte Biomedical Corporation, Rockville, MD, USA) Serum cotinine-ID HPLC-APCI MS/MS	Two-sided student *t*-tests Wald chi-square analysis Multivariate linear regression models (evaluate the associations between HIV status and each heavy metal). Statistical analysis performed using SAS Institute Inc., Cary, NC, USA)
Obirikorang et al., 2016	Comparative cross-sectional	HAART-treated (219), HAART-naïve (100)	Blood (Serum)	CD4/CD3 lymphocyte count-flow cytometry by flow cytometry (BD FACSCOUNT, Becton Dickenson and Company, San Diego, CA, USA) haemoglobin and white cell indices. (Mindray BC 3000 Plus Mind ray Company, Shenzhen, China). serum iron, ferritin, transferrin, and transferrin saturation (TSAT)-Flexor XL analyzer from vital scientific serum CRP-semi quantitative immune-chromatographic method	Unpaired *t*-test (compare means of continuous variables), Fisher’s exact test/chi-square, one-way ANOVA. Data analyzed using Graph pad Prism version 6.0 for windows (Graph pad software, San Diego, CA, USA).
Li et al., 2017	Cross-sectional	HIV positive men (50)	serum plasma, urine, semen	Seminal, plasma, and urine Cd, Pb, Zn-atomic absorption spectrophotometer (Perkinelmer analyst400, Perkinelmer, Inc., Waltham, MA, USA) Cd and Pb are detected by graphite furnace atomic absorption spectrophotometer FSH, LH, testosterone-unicel dxl 800 analyzer (Beckman Coulter, Fullerton, CA, USA). HIV RNA viral load- HIV viral load kit (Roche, Indianapolis, In) and Cobas Taqman HIV test. Semen- Weili semen analyzer (wljx 9000, Beijing Weili New Century Technology Development Co., Ltd., Beijing, China)	ANOVA Spearman’s rank correlation data analyzed using SPSS 16.0 for windows (SPSS Inc., Chicago, IL, USA)
Ma, et al., 2018	Cross-sectional	HIV HAART-treated (100), HIV HAART-naïve (100), HIV-negative Controls (100)	Blood(Plasma)	HIV Screening-Unigold, Determine, CD-4 T count- Cyflow counter flow cytometer (Facs Flow Cytometer count system, Lincolnshire, IL, USA). Plasma levels of Pb, Cd, Hg, and Ni- Inductively Coupled Plasma Mass Spectrometer (ICP-MS), Agilent 7500, Norwalk, CT, USA.	Student’s *t*-test, ANOVA, Pearson’s correlation coefficient Statistical software SPSS version IBM 21 (SPSS Inc., Chicago, IL, USA)
Wiraguna et al., 2019	Cross-sectional	HIV positive (18), HIV negative (27)	Blood (Plasma)	Zn-Spectrophotometer (inductively coupled plasma mass spectrometry). CD4 count-flow cytometry. HIV screening-Ongkoprob Intec rapid test with ST Biolin reagents (USA)	Bivariate analysis, chi-square test, unpaired *t*-test, Shapiro–Wilk, SPSS 23.0
Barocas et al., 2019	Cross-sectional	HIV positive (204)	Blood (Plasma)	Zn level testing (ImmunoBioService laboratory, St. Petersburg) Laboratory assays: ALT, AST, platelet count (St. Petersburg Pasteur Institute Central Clinical Diagnostic Laboratory, Northwestern Federal District, Russia) liver stiffness-Elastography (Fibro scan)	Generalized additive models (GAMs), Multiple linear regression models, chi-square, Fisher’s exact test (comparison of groups for categorical variables), two-tailed tests *t*-tests and Wilcoxon tests (for continuous variables) Analysis performed using SAS version 9.3 (SAS Institute, Inc., Cary, NC, USA)

HIV, human immunodeficiency virus; EIA, enzyme immunoassay; HPLC, high-performance liquid chromatography; APCI, atmospheric pressure chemical ionization; MS, mass spectrometry; CD4, cluster of differentiation 4; CD3, cluster of differentiation 3; TSAT, transferrin saturation; CRP, C-reactive protein; Cd, cadmium; Pb, lead; Zn, zinc; FSH, follicle-stimulatinghormone; LH, luteinizing hormone; RNA, ribonucleic acid; AST, aspartate aminotransferase.

**Table 3:** Study Findings Stratified by Heavy Metal Markers.

Heavy Metal Marker	Author	Sample Size	Assessment Method	Findings	Outcome Measured/Method	Associations between Heavy Metal Markers and Outcome Measured
Lead (Pb)	Xu et al., 2013	11,761	ICP-MS	HIV:1.43 (1.17–1.75), Non-HIV Negative:1.11 (1.09–1.14) *p* = 0.02	Elevated prevalence of heavy metals in HIV patients	Pb levels were higher in HIV-infected patients aged 18–34; who reported being neither Hispanic, white, or black; who had only graduated from high school compared to non-HIV, *p* < 0.05. Female subjects with HIV infection had higher levels of blood lead compared to females without HIV.
	Li et al., 2017	50	graphite furnace atomic absorption spectrophotometer	Seminal Pb: 8.57 ± 0.86 μg/L Urine Pb: 5.34 ± 0.41 μg/L Serum Pb: 6.40 ± 0.45	Effects on reproductive parameters: FSH, LH, Testosterone	HIV-1 viral loads were significantly associated with increased seminal Pb. Seminal Pb positively correlated with LH; Serum Pb negatively correlated with FSH.
	Ma et al., 2018	300	ICP-MS	HIV-Positive: 1.22 ± 1.00 HIV-Negative: 0.57 ± 0.41 HAART-Naive: 1.07 ± 0.85 μg/dL HAART-Treated: 1.38 ± 1.16 HIV-Neg Controls: 0.57 ± 0.41 μg/dL *p* < 0.001	Comparison of heavy metal concentration in HIV-Positive HAART-treated and HIV-Positive HAART-naive	Blood level of Pb decreased with increasing CD4 count.
Cadmium (Cd)	Xu et al., 2013	11,761	ICP-MS	HIV:0.47 μg/dL (0.38–0.59) Non-HIV:0.34 μg/dL (0.33–0.35) *p* < 0.01	Elevated prevalence of heavy metals in HIV patients	HIV individuals had higher Cd levels compared with control. Female subjects with HIV infection had higher levels of blood Cd compared to females without HIV. Cd blood level was higher in male HIV-infected subjects aged 35 to 49.
	Li et al., 2017	50	graphite furnace atomic absorption spectrophotometer	Seminal Cd: 1.69 ± 0.33 μg/dL Urine Cd: 1.41 ± 0.17 μg/dL Serum Cd: 0.33 ± 0.44 μg/dL	Effects on reproductive parameters: FSH, LH, Testosterone	Seminal Cd negatively correlated with motile sperm and motile sperm rate and positively correlated with immotile rate and immotile sperm count. Urine Cd was negatively correlated with serum testosterone. Serum Cd was negatively correlated with progressively motile sperm. Cd was significantly correlated with semen quality and serum hormone in HIV-infected samples.
	Ma et al., 2018	300	ICP-MS	HIV Subjects: 0.62 ± 0.27 μg/dL HIV-Neg: 0.10 ± 0.01 *p* < 0.001	Comparison of heavy metal concentration in HIV-Positive HAART-treated and HIV Positive HAART-naive	Blood level of Cd decreased with increasing CD4 count.
				HAART-Naive: 0.55 ± 0.26 μg/dL HAART-Treated: 0.68 ± 0.04 μg/dL HIV-Neg Controls: 0.10 ± 0.01 *p* < 0.001		
Zinc (Zn)	Barocas et al., 2019	204	Zn level testing (ImmunoBioService laboratory, St. Petersburg)	Adjusted Odd’s ratio(95%CI):1.25(0.62–2.53)	Impact of Zn deficiency on occurrence of liver fibrosis among ART-naive young HIV/HCV co-infected persons	No significant association was found between continuous zinc level and FIB-4 score.
	Wiraguna et al., 2019	45	ICP-MS	HIV-Infected with CA: 57.27 ± 8.32 HIV Non-Infected with CA: 64.59 ± 8.20 *p* = 0.006	Comparison of mean plasma Zn levels in condyloma acuminata patients with HIV and without HIV infection.	The mean plasma Zn levels in condyloma acuminata patients with HIV were significantly lower than those without HIV infection.
Mercury (Hg)	Xu et al., 2013		ICP-MS	HIV:1.04 μg/dL(0.69–1.55) Non-HIV:0.91 μg/dL (0.86–0.96) *p* = 0.50	Elevated heavy metal concentration in HIV patients	Subjects with HIV had significantly higher but not statistically significant different levels of total Hg (1.04 vs. 0.91 μg/dL, *p* = 0.5) than HIV-uninfected population.
	Ma et al., 2018	300	ICP-MS	HIV-Positive: 0.08 ± 0.00 μg/dL HIV-Negative: 0.04 ± 0.00 μg/dL *p* < 0.001 HAART-Naive: 0.06 ± 0.02 μg/dL HAART-Treated: 0.09 ± 0.01 μg/dL HIV-Negative Controls: 0.04 ± 0.00 μg/dL *p* < 0.001	Comparison of heavy metal concentration in HIV-positive HAART-treated and HIV-positive HAART-naive	Mean blood levels of Hg in HIV-positive subjects was significantly higher than in the control subjects (*p* < 0.001). Blood level of Hg decreased with increasing CD4 count.
Iron (Fe)	Obirikorang et al., 2016	319	Flexor XL analyzer from vital scientific	Fe(μmol/L): Total−13.63 ± 11.73, HAART-Treated: 14.51 ± 12.40 HAART-Naive: 9.70 ± 3.94 *p* = 0.0187 Ferritin(μg/L): Total: 255 ± 51.48 HAART-Treated: 265.202 ± 89.96HAART-Naive: 238.10 ± 57.45 *p* = 0.0691 Transferrin (mg/dL): Total:203.90 ± 36.81, HAART-treated:199.60 ± 30.28, HAART-Naive: 223.20: ± 54.05 *p* = 0.0002 TIBC(dL): Total: 259 ± 46.75HAART-Naive: 253.50 ± 38.45 *p* = 0.0002 %TSAT: Total:30.82 ± 27.08, HAART-Treated:33 ± 18.57 HAART-Naive:21.06 ± 10.85 *p* = 0.0114	To determine the prevalence of anaemia and evaluated markers of iron homeostasis in a cohort of HIV patients	Serum iron(*p* < 0.0019), ferritin (*p* < 0.0021), and TSAT (*p* < 0.0002) were significantly higher in anaemic than non anaemic patients. Serum transferrin (*p* < 0.0001) and TIBC (*p* < 0.0001) were, however, higher in anaemic than non-anaemic patients. Participants with anaemia had a significantly lower CD4/CD3 lymphocyte count (*p* < 0.0001). Serum ferritin(*p* = 0.9022),transferrin (*p* = 0.0143), and TIBC (*p* = 0.0143).
Nickel (Ni)	Ma et al., 2018	300	ICP-MS	HIV-Positive:0.89 ± 1.19 μg/dL HIV Negative:0.11 ± 0.01 *p* < 0.001 HAART-naive:0.95 ± 1.51 μg/dL HAART-treated: 0.84 ± 0.11 μg/dL HIV-Negative Controls: 0.11 ± 0.01 *p* < 0.001	Comparison of heavy metal concentration in HIV-positive HAART-treated and HIV-positive HAART-naive	Mean blood levels of nickel were significantly higher in HIV-positive patients compared to controls. Ni level increased with increasing CD4 count but without statistical significance.

HIV, human immunodeficiency virus; Pb, lead; Cd, cadmium; Zn, zinc; Hg, mercury; Ni, Nickel; Fe, iron; CA, condyloma acuminata; HCV, hepatitis C virus; FIB-4, fibrosis-4; TIBC, total iron-binding capacity; TSAT, transferrin saturation; ICP-MS, inductively coupled plasma-mass spectrometry; CD4, cluster of differentiation 4; FSH, follicle-stimulating hormone; LH, luteinizing hormone; ART, antiretroviral therapy.

## Data Availability

All data relevant to the study are included in the article. Data are collected from the studies published online, publicly available, and specific details related to data will be made available upon request.
